# Sterility Caused by Floral Organ Degeneration and Abiotic Stresses in *Arabidopsis* and Cereal Grains

**DOI:** 10.3389/fpls.2016.01503

**Published:** 2016-10-14

**Authors:** Ashley R. Smith, Dazhong Zhao

**Affiliations:** Department of Biological Sciences, University of Wisconsin-Milwaukee, MilwaukeeWI, USA

**Keywords:** sterility, yield, floral organ degeneration/abortion, ABC genes, hormones, abiotic stresses, *Arabidopsis*, cereal crops

## Abstract

Natural floral organ degeneration or abortion results in unisexual or fully sterile flowers, while abiotic stresses lead to sterility after initiation of floral reproductive organs. Since normal flower development is essential for plant sexual reproduction and crop yield, it is imperative to have a better understanding of plant sterility under regular and stress conditions. Here, we review the functions of ABC genes together with their downstream genes in floral organ degeneration and the formation of unisexual flowers in *Arabidopsis* and several agriculturally significant cereal grains. We further explore the roles of hormones, including auxin, brassinosteroids, jasmonic acid, gibberellic acid, and ethylene, in floral organ formation and fertility. We show that alterations in genes affecting hormone biosynthesis, hormone transport and perception cause loss of stamens/carpels, abnormal floral organ development, poor pollen production, which consequently result in unisexual flowers and male/female sterility. Moreover, abiotic stresses, such as heat, cold, and drought, commonly affect floral organ development and fertility. Sterility is induced by abiotic stresses mostly in male floral organ development, particularly during meiosis, tapetum development, anthesis, dehiscence, and fertilization. A variety of genes including those involved in heat shock, hormone signaling, cold tolerance, metabolisms of starch and sucrose, meiosis, and tapetum development are essential for plants to maintain normal fertility under abiotic stress conditions. Further elucidation of cellular, biochemical, and molecular mechanisms about regulation of fertility will improve yield and quality for many agriculturally valuable crops.

## Introduction

**FIGURE 1:**
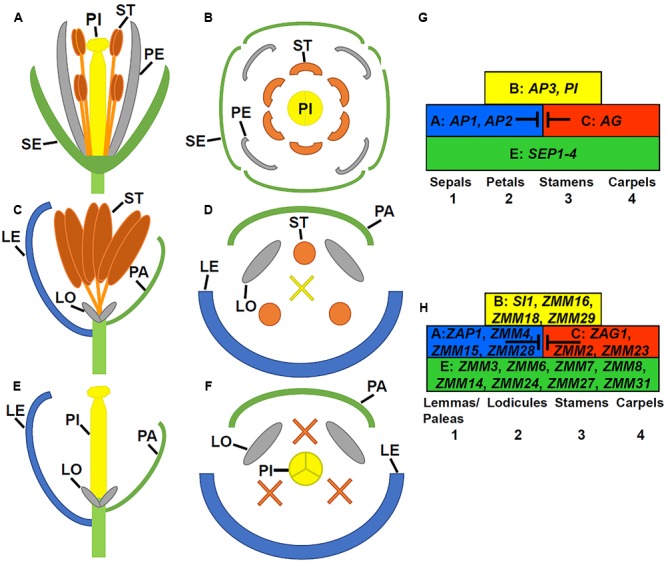
**Flower structures in *Arabidopsis* and maize as well as ABCE model. (A)** A longitudinal view through a mature *Arabidopsis* flower (only 2 of 4 long stamens shown). **(B)** A cross view through an *Arabidopsis* flower. **(C)** A longitudinal view through a mature male upper floret in maize. **(D)** A cross view through a male maize flower. **(E)** A longitudinal view through a mature female upper floret in maize. **(F)** A cross section view a female maize flower. LE, lemma; LO, lodicule; PA, palea; PE, petal; PI, pistil; SE, sepal; and ST, stamen. X indicates the aborted carpels. **(G)** The ABCE model in *Arabidopsis*. **(H)** The ABCE model in maize with most likely orthologous genes.

**FIGURE 2:**
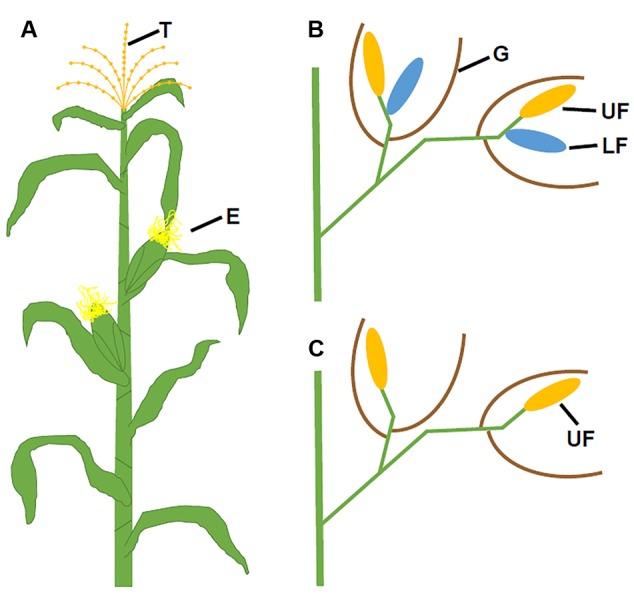
**Positions of maize florets in ear and tassel. (A)** A mature maize plant with positions of the tassel and ears. **(B)** Zoomed in view of male florets in the tassel showing the upper florets (yellow), lower florets (blue), and glumes (brown). **(C)** Enlarged view of female florets in the ear showing only the upper florets (yellow) after the degeneration of the lower florets. E, ears; G, glumes; LF, lower floret, T, tassel; and UF, upper floret. **(B,C)** Modified from Bortiri and Hake (2007).

Flower development is a long and complex process, which is mainly classified into four stages: flowering transition, floral meristem identity, floral organ identity, and floral organ morphogenesis. Mainly using model species *Arabidopsis thaliana* and snapdragon (*Antirrhinum majus*), extensive molecular genetic studies have identified numerous genes required for flower development, particularly during early stages. *Arabidopsis* plants produce raceme-type indeterminate inflorescences where flowers are indefinitely generated. A typical *Arabidopsis* flower contains four protective sepals in the first whorl, four petals in the second whorl, six stamens (male reproductive organs) in the third whorl, and two fused carpels (female reproductive structure) that form the gynoecium in the fourth whorl (**Figures 1A,B**). Different from *Arabidopsis*, Poaceae plants, commonly known as grasses, produce determinate panicles where flowers (or florets) are organized into spikelets. In maize, these spikelets are grouped into separate male and female inflorescences (**Figure 2A**). The highly branched male inflorescence, the tassel, is composed of spikelet pairs, each of which comprises an upper and a lower floret surrounded by the leaf like structures known as glumes (**Figure 2B**). Similarly, spikelet pairs are formed in the female inflorescence, but the lower floret in each spikelet pair is aborted (**Figure 2C**) (Zhang and Yuan, 2014). Poaceae flowers have stamens and carpels similar to eudicot flowers, such as *Arabidopsis*. In maize, the male floret contains three stamens (**Figures 1C,D**), while the female floret produces three central carpels which are fused to form the pistil (**Figures 1E,F**) (Zhang and Yuan, 2014). Maize florets do not contain sepals and petals. Instead, the sepal-analogous organs lemma and palea are produced (**Figures 1C–F**) (Schmidt and Ambrose, 1998; Lombardo and Yoshida, 2015). Additionally, the petal analogous structures known as lodicules are essential for pollination via opening the bract organs (Yoshida, 2012).

Within cereal grains, floral organ degeneration is not unique to maize. During development in grain crops such as wheat (*Triticum aestivum*), rice (*Oryza sativa*), and sorghum (*Sorghum bicolor*), the arrest of stamen or carpel primordia, or both potentially results in reduced fertility or completely sterile flowers (Bommert et al., 2005; Yoshida and Nagato, 2011; Aryal and Ming, 2014). In the floret pair of sorghum, one floret is bisexually fertile, whereas the other one is bisexually sterile. Similarly, wild barley (*Hordeum vulgare*) produces a central fertile floret surrounded by a pair of sterile florets, and even oats (*Avena sativa*) are known to form sterile flowers at the apex of the rachilla (Schmidt and Ambrose, 1998). Additionally, abiotic stresses cause flower sterility, which consequently results in yield loss. In this review, we will focus on discussing molecular genetic and physiological mechanisms underlying sterility caused by floral organ degeneration and abiotic stresses mainly in *Arabidopsis* and key cereal grain plants.

## Molecular Genetic Regulation of Floral Organ Degeneration

Degeneration or abortion of developing stamens and/or pistil is a main mechanism used by plants to produce unisexual flowers or sterile flowers. In *Arabidopsis*, there are four major classes of genes that specify floral organ identity. Class A genes [*APETALA1* (*AP1*) and *AP2*], class B genes [*AP3* and *PISTILLATA (PI*)], the class C gene [*AGAMOUS* (*AG*)], and the semi-redundant class E genes [*SEPALATTA1-4* (*SEP1-4*)]. Class A and E genes are required for specifying sepals in the first whorl. Class A, B, and E genes in combination control petal identity in the second whorl. Class B, C, and E genes direct the stamen identity in the third whorl, and class C and E genes specify carpels in the fourth whorl (**Figure 1G**) (Bowman et al., 1991; Rounsley et al., 1995; Pelaz et al., 2000; Ditta et al., 2004). The ABCE model can also be applied to flower development in other plants including the Poaceae, although many variations exist (**Figure 1H**; **Table 1**). Altered expression patterns of B and C class genes can result in floral organ degeneration and sterility.

**Table 1:** Key genes for maize unisexual flower development.

Gene	Function	Reference
*ZAP1*	Class A function	Mena et al., 1995
*ZMM4*	Class A function	Fischer et al., 1995
*ZMM15, ZMM28*	Class A function	Münster et al., 2002
*IDS1*^∗^, *SID1*^∗^	Class A function	Chuck et al., 1998, 2008
*SI1*	Class B function	Ambrose et al., 2000; Whipple et al., 2004
*ZMM16, ZMM18, ZMM29*	Class B function	Münster et al., 2001; Whipple et al., 2004
*ZMM2*	Class C function	Theissen et al., 1995
*ZMM23*	Class C function	Münster et al., 2002
*ZAG1*	Class C function	Schmidt et al., 1993
*ZAG2*	Class D function	Schmidt et al., 1993
*ZMM1*	Class D function	Theissen et al., 1995
*ZMM25*	Class D function	Münster et al., 2002
*ZAG3*	*AGL6* function	Mena et al., 1995; Thompson et al., 2009
*ZMM6*	Class E function	Fischer et al., 1995
*ZMM24, ZMM27, ZMM31*	Class E function	Münster et al., 2002
*ZmLHS1a, ZM1HS1b (ZMM8, ZMM14)*	Class E function	Cacharrón et al., 1999
*ZMM3*	Class E function	Fischer et al., 1995; Kobayashi et al., 2010
*ZMM7*	Class E function	Fischer et al., 1995
*TS1*	*TS2* expression and JA production	Calderon-Urrea and Dellaporta, 1999
*TS2, TS4, TS6*	Pistil abortion	DeLong et al., 1993; Irish, 1997
*SK1*	Protects pistils	Calderon-Urrea and Dellaporta, 1999
*RMR6*	Protects tassel by repressing *SK1*	Parkinson et al., 2007
*NA1*	Promotes stamen development, BR production	Hartwig et al., 2011
*OPR7, OPR8*	Promote carpel abortion and stamen development; JA production	Yan et al., 2012
*AN1*	promotes stamen arrest; GA production	Bensen et al., 1995
*D1, D2, D3, D5*	Promote stamen arrest; GA production	Fujioka et al., 1988; Spray et al., 1996

*^∗^Cannot compensate for the *AP2* mutation in *Arabidopsis*.*

The extensive roles and interactions of ABC genes in flower development are summarized in Prunet and Jack (2014). What is less clear and receives less attention is that after the establishment of floral organ identity, how floral organ identity genes play a role in development of functional floral organs. *AG* is required throughout reproductive development for establishing fertility. Specifically, *AG* is expressed in stamen and carpel primordia initially, and later in specific cell types of stamens and carpels (Bowman et al., 1991). AG (along with PI and AP3) controls stamen development via directly activating the expression of *SPOROCYTELESS/NOZZLE* (*SPL/NZZ*), which in turn regulates microsporogenesis (Ito et al., 2004; Liu et al., 2009). AG also upregulates the expression of the *DEFECTIVE IN ANTHER DEHISCENCE 1* (*DAD1*) gene that encodes a jasmonic acid (JA) biosynthesis enzyme (Ito et al., 2007). The *dad1* mutant produces immature pollen, resulting in male sterility. If *AG* is not expressed in flowers prior to stage 7 in *Arabidopsis*, plants fail to undergo microsporogenesis, while increased duration of *AG* expression enhances normal stamen and pollen production (Ito et al., 2007). Similarly, in maize *branched silkless1-2* (*bd1-2*) mutants, loss of expression of class C and D (ovule specification) genes like *ZAG1* (*Zea mays AG1*), *ZAG2*, and *ZMM2* (*Zea mays MADS2*) may cause female sterility (Colombo et al., 1998).

Flowers destined to be male or female often begin as hermaphroditic flowers, but later undergo a programmed degeneration of the gynoecium or androecium, respectively, in early reproductive development. This degeneration is often accompanied by down regulation of B and C class genes (Ainsworth et al., 2005). Unisexual flowers in plants like asparagus (*Asparagus officinalis*) undergo abortion late in development at the onset of meiosis, although remnants of male or female organs remain (Dellaporta and Calderon-Urrea, 1993; Aryal and Ming, 2014). In female asparagus flowers, the expression of B class gene *AODEF* (*Asparagus officinalis DEFICIENS*) is decreased in the stamen, which may cause stamen degeneration (Park et al., 2003). Loss of class B gene function also leads to stamen degeneration in the tulip (*Tulipa gesneriana*) mutant *viridiflora* (Kanno et al., 2007). In male sorrel (*Rumex acetosa*) flowers, both class B and C genes are present in early male flower formation. In later stages, the expression of a class C gene is not detectable in the region that would specify carpels in a female or hermaphroditic flower (Ainsworth et al., 2005). In white champion (*Silene latifolia*), the class C gene *SLM1* (*Silene latifolia MADS1*) is expressed until meiosis in male and female floral organs. Later in female flower development, stamens undergo degeneration. The expression of *SLM1* is not detected in aborted stamens, while its expression persists in the undeveloped gynoecium of male flowers (Hardenack et al., 1994). Moreover, in *S*. *latifolia, Arabidopsis* orthologs of *SHOOT MERISTEMLESS* (*SLSTM1* and *SLSTM2)* and *CUP SHAPED COTYLEDON* (*SLCUC1* and *SLCUC2*) likely control sex determination via regulating cellular proliferation in the third whorl (Zluvova et al., 2006).

Growing evidence supports that the early loss of class B and C genes leads to the arrest of development in reproductive organ primordia and ultimately the inability of these flowers to form functional carpels or stamens. It is clear that during development class B and C genes must be expressed in the correct location for a sufficient duration. Without the normal expression, flowers exhibit a wide array of phenotypes, ranging from floral organs present in incorrect whorls, to unisexual flower development, and even complete sterility.

Besides class B and C genes, many additional genes are essential for the establishment of the unisexual state in monoecious plants. Male and female flower development in plants like maize begins as identical, but degeneration of gynoecium primordia in the male flowers and degeneration of stamen primordia in the female flowers result in the production of two distinct flower types (Dellaporta and Calderon-Urrea, 1993; Irish, 1996). *TASSEL SEED* (*TS*) genes are responsible for normal pistil abortion in the tassel. In recessive *ts1* and *ts2* mutants, feminization of tassels occurs and pistillate flowers are formed. The *ts1* mutant phenotype is attributable to a mutation in a lypoxygenase that produces JA (DeLong et al., 1993; Malcomber and Kellogg, 2006; Acosta et al., 2009). *TS2* (a short-chain alcohol dehydrogenase) triggers the programmed cell death (PCD) of pistils (DeLong et al., 1993; Parkinson et al., 2007). In *silkless1* (*sk1*) mutants, pistils are not developed in female florets, while male florets are unaffected (Malcomber and Kellogg, 2006). In the ear, *SK1* protects pistils from undergoing PCD caused by *TS2* (Calderon-Urrea and Dellaporta, 1999). Similarly, in the maize relative *Tripsacum*, the *TS2* homolog *GYNOMONOECIOUS SEX FORM1* is expressed in pistils prior to abortion (Li et al., 1997).

Moreover, in *required to maintain repression6* (*rmr6*) mutants, pistils fail to abort, which causes the feminization in tassels (Parkinson et al., 2007). *RMR6* (encoding the largest subunit of RNA polymerase IV, an orthologue of *Arabidopsis* NRPD1a) acts by limiting the activity of *SK1* to the primary ear floret, resulting in PCD of the gynoecium in the tassel and the secondary ear floret (Parkinson et al., 2007; Erhard et al., 2009). In each of the dominant single mutants of *Ts3, Ts5*, and *Ts6*, as well as the recessive mutant *ts4*, a variety of phenotypes are observed in the tassel, such as reduced tassel size, bisexual flowers, and feminization of the tassel (Veit et al., 1993; Irish et al., 1994). Key genes involved in unisexual flower development in maize are summarized in **Table 1**.

In addition to the formation of unisexual flowers, completely sterile flowers are also commonly produced in grasses due to floral organ degeneration. In some cereal grains, a fertility conversion of sterile flowers is possible. In the sorghum *multiseeded1* (*msd1*) mutant, the development of bisexually sterile flowers become normal, leading to the formation of all fertile flowers (Burow et al., 2014). In barley, the *vrs1* (*six-rowed spike1*) mutation results in fully fertile barley known as six-rowed barley. In the wild-type barley, the *VRS1* gene suppresses lateral spikelet development, causing a central fertile floret surrounded by two sterile florets (Komatsuda et al., 2007).

## Roles of Hormones in Floral Organ Development

**FIGURE 3:**
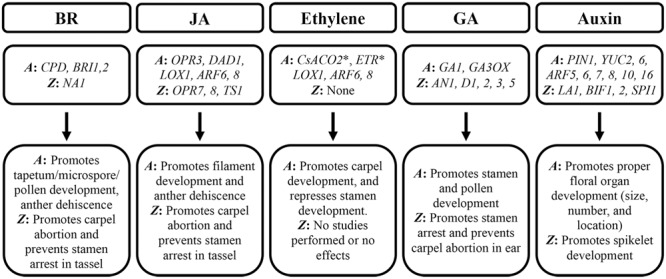
**Effects of hormones on male and female flower development.** Genes involved in hormone biosynthesis, transport, and perception are shown. *A*: *Arabidopsis thaliana, Z*: *Zea mays*, ^∗^ Indicates cucumber genes used in *Arabidopsis* studies.

Hormones have strong influences on flower sexuality and fertility. Some hormones are essential for both male and female organ development, while others are male or female specific (**Figure 3**). Brassinosteroids (BRs) and JA promote male but suppress female organ development in both *Arabidopsis* and maize (Clouse et al., 1996; Szekeres et al., 1996; Clouse and Sasse, 1998; Stintzi and Browse, 2000; Zhao and Ma, 2000; Li et al., 2001; Nagpal et al., 2005; Mandaokar et al., 2006; Acosta et al., 2009; Yan et al., 2012). Ethylene has been shown to act as a feminizing agent in plants like cucumber, but its role in *Arabidopsis* and maize is less understood (Yin and Quinn, 1995; Duan et al., 2008; Wang et al., 2010). The function of gibberellic acid (GA) is conflicting, as it is critical for proper male organ development in *Arabidopsis*, but it antagonizes stamen development in the maize ear (Fujioka et al., 1988; Dellaporta and Calderon-Urrea, 1994; Bensen et al., 1995; Spray et al., 1996; Goto and Pharis, 1999; Michaels and Amasino, 1999; Cheng et al., 2004; Yu et al., 2004; Hu et al., 2008). Differently, auxin is necessary for both male and female floral organ development (Okada et al., 1991; Sessions et al., 1997; Nagpal et al., 2005; Cheng et al., 2006; Wu et al., 2006; Dong et al., 2013).

### Brassinosteroid

Brassinosteroids are widely involved in cell expansion, cell division, senescence, vascular differentiation, and stress responses. Overall, BRs promote the formation of stamens and pollen in both *Arabidopsis* and maize, and the abortion of pistils in staminate maize flowers. The *constitutive photomorphogenesis and dwarfism* (*cpd*) mutant which fails to form the ecdysone*-*like brassinosteroids, produces pollen defective in pollen tube elongation (Szekeres et al., 1996). Both *cpd* and *brassinosteroid-insensitive1* (*bri1*) mutants make far fewer pollen grains per locule with limited viability. Similarly, the *brassinosteroid-insensitive2* (*bin2*) mutant is male sterile (MS; Li et al., 2001). Further studies show that BRs control male fertility via regulating expression of genes critical for anther and pollen development, such as *SPL/NZZ, DEFECTIVE IN TAPETAL DEVELOPMENT AND FUNCTION 1* (*TDF1*), *ABORTED MICROSPORES* (*AMS*), and *MS1* and *MS2* genes (Ye et al., 2010). Additionally, BRs are required for sex determination in maize. In the maize *nana plant 1* (*na1*) mutant tassel, some carpels fail to abort, resulting in both staminate and pistillate flowers (Hartwig et al., 2011). The *na1* mutation occurs in *ZmDET2*, a homolog of the *Arabidopsis DE-ETIOLATED2* (*DET2*) which encodes the important BR biosynthesis enzyme 5α-steroid reductase, suggesting an important role of BRs in the formation of tassel flowers in maize.

### Jasmonic Acid

In *Arabidopsis* and the maize tassel, JA is crucial for stamen and pollen maturation. In *Arabidopsis*, the *12-oxophydoienoic acid reductase 3* (*opr3*) mutant is deficient in JA synthesis at the conversion of linolenic acid to JA. The *opr3* mutant produces stamens that are abnormal in filament elongation and dehiscence (Stintzi and Browse, 2000; Zhao and Ma, 2000; Mandaokar et al., 2006). Maize has a series of *OPR* genes, among which *OPR7* and *OPR8* represent the *Arabidopsis OPR3* orthologs (Zhang et al., 2005; Yan et al., 2012). The *opr7-5 opr8-2* double mutant plants form feminized tassels devoid of stamen formation and are capable of seed production if pollinated with wild-type pollen. This phenotype can be reversed by exogenous application of JA (Acosta et al., 2009; Yan et al., 2012). A similar phenotype is observed when the *AG* expression is lost, as AG promotes the *DAD1* (JA biosynthesis enzyme) expression (Ito et al., 2007). AUXIN RESPONSE FACTOR (ARF) transcription factors ARF6 and ARF8 are required for JA production. Disruption of *ARF6* and *ARF8* genes results in delayed stamen development, and consequently the complete male sterility (Nagpal et al., 2005). As discussed above, feminization in the *ts1* tassel is attributed to loss of JA synthesis (Acosta et al., 2009). In the tomato *JAjas–insensitive1* (*jai-1*) mutant, the male fertility is also greatly affected with about 28% of pollen being viable and only 10% actually germinating (Li et al., 2004). However, it is believed that the additional female sterility may be caused by arrest in embryo/seed development.

The effects of JA on stamen development and male fertility in maize and *Arabidopsis* are consistent. In both organisms JA promotes male organ development, but suppresses female organ development. Unlike in *Arabidopsis*, JA is also important for female fertility in tomato, indicating potentially divergent and complex roles of JA in plant sexual reproduction that warrant further exploration. Of particular interest, AG is required for the *DAD1* expression, suggesting interaction between the JA signaling and the class C gene *AG*.

### Ethylene

Ethylene promotes the formation of female flowers in cucumber (*Cucumis sativus*). *CsACO2* (*OXIDASE GENE2*) encodes an ACC OXIDASE which oxidizes ethylene intermediates to form ethylene. Transgenic *Arabidopsis* plants expressing *CsACO2* under control of the *AP3* promoter display repressed stamen development and male sterility (Yin and Quinn, 1995; Duan et al., 2008). Down-regulation of the ethylene receptor gene *ETR1* results in the decrease of the ETR1-interacting kinase CTR1, which a repressor of the ethylene signaling. Loss of ETR1 fails the formation of ETR1-CTR1 complex. Consequently, de-suppression of the ethylene response pathway causes the production of female flowers in *Arabidopsis* (Wang et al., 2010). So far, little is known about the effects of ethylene on flower development in monocots, including maize.

### Gibberellin

In *Arabidopsis*, gibberellin (GA) deficiencies greatly impact male fertility, resulting in partial or complete male sterility. Conversely, GA deficiencies promote stamen maturation in the maize ear, leading to the formation of perfectly bisexual flowers.

In *Arabidopsis*, the GA deficient mutant *ga1-3*, which fails to catalyze the first step in GA biosynthesis due to a deletion in ent-KAURENE SYNTHASE, exhibits abnormal microsporogenesis and retarded growth of all floral organs, e.g., stamens with greatly shortened filaments that cannot pollinate pistils (Michaels and Amasino, 1999; Cheng et al., 2004; Yu et al., 2004). Similarly, the *ga1-1* mutant has severe defects in stamen and pollen maturation as well as petal and sepal growth (Goto and Pharis, 1999). DELLA proteins (transcriptional repressors), such as RGA, RGA-LIKE1 (RGL1), and RGL2, repress stamen development (Cheng et al., 2004). The DELLA degradation triggered by GA activates JA biosynthesis genes *DAD1* and *LIPOXYGENASE 1* (*LOX1*; Cheng et al., 2009).

In *Arabidopsis*, during GA biosynthesis, four *GIBBERELLIN 3-OXIDASE* (*GA3OX*) genes are responsible for the final GA activation. The *ga3ox1 ga3ox3* double mutant shows high frequency of sterility on the lowest siliques with fertility restoration after the 20^th^ to 25^th^ silique, whereas triple mutants *ga3ox1 ga3ox3 ga3ox4* and *ga3ox1 ga3ox2 ga3ox3*, on average, underwent a later conversion. This sterility is caused by abnormal anther dehiscence and shortened anther filaments, highlighting that GA is required for stamen development in *Arabidopsis* (Hu et al., 2008).

In the maize ear, GA promotes the arrest of stamens, but prevents carpel abortion. The maize *ANTHER EAR1* (*AN1*) gene is necessary for the production of ent-kaurene during GA biosynthesis. Besides short stature and delayed maturity, the *an1* mutant develops perfectly bisexual flowers in ears, indicating the inability of the *an1* plant to successfully abort stamens in the ear (Bensen et al., 1995). In addition, maize dwarf mutants *d1, d2, d3*, and *d5*, which are deficient in GA production, also form stamens in flowers of the ear (Fujioka et al., 1988; Dellaporta and Calderon-Urrea, 1994; Spray et al., 1996).

Taken together, in both dicots and monocots the male organ development is sensitive to GA, however, its effects are opposite.

### Auxin

In *Arabidopsis* and maize, auxin is required for the formation of all floral organs, as disruption of genes associated with auxin signaling, biosynthesis, and transport leads to flowers with various abnormalities (Okada et al., 1991; Nagpal et al., 2005; Cheng et al., 2006; Wu et al., 2006; Cecchetti et al., 2008). ARFs activate or repress expression of auxin response genes. In *Arabidopsis*, the *arf6 arf8* double mutant and plants expressing miR167 resistant versions of *ARF6* and *ARF8* exhibit many flower defects, such as shortened petals, gynoecium, and stamen filaments, failure to release pollen, as well as abnormal ovules (Nagpal et al., 2005; Wu et al., 2006). In the *arf3*/*ett* (*ettin*) mutant, a decreased number of stamens are observed (Sessions et al., 1997). In the *Arabidopsis floral organs in carpels* (*foc*) mutant, increased expression of *ARF10, 16*, and *17* due to the lack of its negative regulator miR160 results in floral organ loss and abnormal female fertility (Liu et al., 2010). In rice, expressing the miR160 resistant version of *OsARF18* causes the formation of abnormal flowers and reduced seed set (Huang et al., 2016a). Mutations in *arf5*/*mp* (*monopteros*) lead to either small or absent lateral flowers (Przemeck et al., 1996). *YUCCA* (*YUC*) genes in *Arabidopsis* encode auxin biosynthesis enzymes. Stamens in the *yuc2yuc6* double mutant fail to elongate but produce pollen grains, while flowers in the *yuc1yuc4* double mutant cannot form functional reproductive organs (Cheng et al., 2006). Ectopic expression of the small protein ligand TAPETUM DETERMINANT1 (TPD1) causes abnormal ovule and seed development via altering auxin signaling (Huang et al., 2016b,c). In maize, mutation in the *SPARSE INFLORESCENCE1* (*SPI1*) gene, which functions as a *YUC*-like gene, results in tassels with small ears and few kernels (Gallavotti et al., 2008a).

Many genes in the auxin transport pathway play key roles in maintaining fertility and normal floral organ development. In *Arabidopsis*, PIN-FORMED (PIN) transporters function in polar auxin transport. In the *pin1*-*1* mutant, various phenotypes, such as missing stamens, the formation of sterile pistil-like structures, and abnormal petal shape, are observed (Okada et al., 1991). In maize, *BARREN INFLORESCENCE1* (*BIF1*) and *BARREN INFLORESCENCE2* (*BIF2*) are involved in regulating polar auxin transport. BIF1 likely acts upstream of polar auxin transport via up-regulating the expression of *ZmPIN1a* (Gallavotti et al., 2008b). The tassels and ears in *bif1* and *bif2* mutants have reduced number of spikelets/florets and floral organs, and consequently fewer kernels (McSteen and Hake, 2001; Barazesh and McSteen, 2008; Skirpan et al., 2009). The rice gene *LAZY1* (*LA1*), which encodes a novel grass specific protein, is a negative regulator of polar auxin transport (Li et al., 2007). In maize, the *la1-ref* mutant carries a mutation in the maize ortholog of *LA1* (Dong et al., 2013). Spikelets in *la1-ref* either are not fully developed or undergo abortion especially in the tassel tip. Similarly, in the ear, silk production is decreased and spikelets are aborted in the ear tip (Dong et al., 2013).

The role of auxin in flower development is challenging to interpret. Due to the effects of auxin on the entirety of plant, it is possible that some phenotypes are the consequence of larger changes occurring in the plant. For example, as mentioned above, the *LA1* gene regulates polar auxin transport in *Arabidopsis*. *LA1* also affects plant architecture, since the tiller morphology is altered in mutants like *la1-ZF802* in a pattern known as tiller-spreading (Li et al., 2007). These architectural changes may alter photosynthesis which consequently affects fertility and yield (Wu et al., 2013). Thus, more work should be done to look into specific roles of all involved hormones in flower development and fertility. Effects of hormones on female and male flower development are summarized in **Figure 3**.

## Sterility And Abiotic Stresses

The loss of yield caused by abiotic stresses is partially attributed to defects in flower development. Even a mild or a short-term abiotic stress can cause a significant decrease in fertility. The majority of studies focus on the effects of abiotic stresses, including heat, cold, and drought, on fertility at the morphological level in *Arabidopsis* and cereal grains; however, the molecular mechanisms behind are not clear.

### Heat Stress

Many stages of flower development, particularly the late stages of stamen development, are sensitive to heat stress. In *Arabidopsis* and cereal grains, sensitive stages include meiosis of pollen mother cells (PMC), tapetum development, anther dehiscence/pollen release, anthesis, and fertilization (Dupuis and Dumas, 1990; Kim et al., 2001; Abiko et al., 2005; Oshino et al., 2007; Thakur et al., 2010; Zinn et al., 2010; De Storme and Geelen, 2014). The overall effects of heat stress on male sterility depend on duration, timing, and temperature (Schoper et al., 1986, 1987a,b). The female organ is not as susceptible as the male organ to the heat stress.

The tapetum in the anther is particularly vulnerable to heat stress (Parish et al., 2012). In *Arabidopsis*, the tapetum consists of a monolayer of cells, which surrounds successive stages of microsporocytes, tetrads, microspores, and developing pollen as anther development progresses. Tapetal cells undergo three stages: differentiation, maturation, and PCD. First, the early differentiated tapetal cells secrete the callase enzyme that is required for the release of haploid microspores from meiotic tetrads. Second, mature tapetal cells produce a large amount of specialized non-photosynthetic plastids (elaioplasts) and tapetosomes, which provide lipids, proteins, and sporopollenin essential for pollen wall formation. Finally, tapetal cells are degenerated via PCD, and the remnants are important for the completion of pollen wall formation (McCormick, 1993; Wu and Cheung, 2000; Parish and Li, 2010).

The abnormal tapetum or altered timing of its degeneration causes pollen defects and consequently male sterility. Barley and wheat grown at elevated temperatures (barley: 30–35°C day/20–25°C night, wheat: 30°C for 1–3 days, or varied 30/20°C day/night at meiosis) display precocious tapetum degradation (Saini and Aspinall, 1982; Saini et al., 1984;Abiko et al., 2005; Oshino et al., 2007; Omidi et al., 2014). In rice, tapetal genes like *YY1* and *YY2* are down regulated following heat stress [39/30°C (day/night) for 5 days], affecting tapetum function and consequently pollen viability (Endo et al., 2009). Additionally in rice, male sterility in the thermos-sensitive genic male-sterile (TGMS) line *95850ms* is caused by premature tapetum PCD and consequent pollen grain collapse (Ku et al., 2001, 2003). A recent study shows that the TGMS trait in the *thermosensitive genic male sterile 5 (tms5)* mutant is caused by the loss of function of RNase Z^s1^, which processes mRNAs of three ubiquitin fusion ribosomal protein genes (Ub_L40_) (Zhou et al., 2014). At restrictive temperatures, high level of UbL40 results in abortive pollen and therefore male sterility. *Arabidopsis* plants under heat stress (31 and 33°C) show reduced expression of *YUCCA* genes especially in tapetum and PMC. Inactivation of *YUC2* and *YUC6* leads to decreased male fertility, while which can be reversed by exogenous application of auxin (Sakata et al., 2010, 2014). More work needs to be done to understand the genetic pathways leading to decreased fertility during heat stress, especially the role of auxin in male fertility and tapetum development.

Another sensitive stage is the PMC meiosis. Wheat and rice exposed to high or varied temperatures [wheat: high 30°C (1–3 days), varied 30°/20°C (day/night), rice: 39/30°C (day/night; 5 days)] at and prior to the onset of PMC meiosis exhibit greatly reduced grain set (Saini and Aspinall, 1982; Saini et al., 1984; Endo et al., 2009; Omidi et al., 2014). Impairments in rice PMC division occur even 5°C over the ambient temperature [28.3/21.3°C (day/night)], resulting in decreased pollen production especially in susceptible cultivars (Prasad et al., 2006).

Anther dehiscence, anthesis, and fertilization are sensitive to elevated temperatures too. Heat stress applied to wheat [two-day intervals of 36/31°C (day/night)] from floral emergence to 3 days post anthesis results in male sterility due to abnormal pollen grains (Tashiro and Wardlaw, 1990; Ferris et al., 1998). Similarly, rice that receives a short-term (33.7°C, 1 h) or a long-term heat stress (35°C, 38°C, and 41°C, 5 days) at anthesis display reduced fertility, but with a better fertility when stress was applied before or after anthesis (Satake and Yoshida, 1978; Jagadish et al., 2007). Heat stressed rice [35/25°C (day/night)] has decreased anther dehiscence and pollen count (Das et al., 2014). Pollen germination is also very vulnerable to high temperature stress. When maize tassels and rice spikelets are subjected to high heat stress [maize: 6 h of 40°C, rice: 35/25°C (day/night) or greater for 3 days], the ability of pollen to fertilize the ear is lost, which is attributed to the failure of pollen tube growth (Dupuis and Dumas, 1990; Das et al., 2014). In *Arabidopsis*, disruption of *THERMOSENSITIVE MALE STERILE 1* (*TMS1*), which encodes the heat shock protein HSP40, causes pollen tubes to burst and decreased pollen tube length (Yang et al., 2009).

Although the cause of the heat-induced sterility is not clear, it might be related to heat shock proteins like HSP40 mentioned above (Yang et al., 2009). Mutations in the small heat shock protein gene *BOBBER1* (*BOB1)* result in a range of phenotypes, such as irregular flowers and sterile siliques (Perez et al., 2009). In maize, pollen infertility may be due to the lack of production of major protective HSPs (Dupuis and Dumas, 1990; Hopf et al., 1992), supported by the fact that pollen grains do not express *HSP* RNAs at dehiscence (Dietrich et al., 1991; Young et al., 2001). In wheat, heat-stress induces many HSPs, including HSP17, HSP26, and HSP70, as well as microRNAs targeted *HSP* genes (Kumar et al., 2015).

### Cold Stress

Extensive research has been done about the effects of below optimal temperature conditions on growth and development of *Arabidopsis* and cereal crops. In *Arabidopsis*, a large number of genes are identified with differential expression after chilling stress, and many of which play roles in pollen development (Lee et al., 2005; Zou and Yu, 2010). The *COLD REGULATED* (*COR*) genes are induced at low temperatures. The WRKY transcription factors repress *COR* expression via binding to their c-repeat binding factors (CBF; Zou et al., 2010). Plants harboring mutated *WRKY* genes show increased pollen viability under the chilling stress. In *Arabidopsis*, freezing treatment (0°C for 72 h) induces the acclimation of COR, lipid transfer proteins, and β-amylase in vegetative tissues, but not in pollen, which may explain the inability of pollen to withstand the chilling stress (Lee and Lee, 2003). In rice, DEHYDRATION RESPONSIVE ELEMENT BINDING PROTEIN1F (*Os*DREB1F) activates the expression of *COR15a*. Overexpression of *OsDREB1F* causes increased cold and drought tolerance, which aids spike development, further highlighting the role of *COR* genes in cold tolerance (Wang et al., 2008). For a review on freezing tolerance genes Thomashow (1999).

In cereal grains, the establishment of reproductive development, branching, and spikelet pair formation are sensitive to low temperature stress. Maize plants grown at cold conditions (10°C for 3 days or longer) during the reproductive transition produce less tassel branches and spikelet pairs (Bechoux et al., 2000). In the maize inbred line Dent11, chilling stress [17/6°C (day/night)] leads to the reduction of 43 and 29% of pollen when stress is applied at branch and spikelet initiation, respectively (Tranel et al., 2009).

In anthers, meiosis and tapetum development are particularly cold sensitive. Sorghum and rice display male sterility under cold conditions during meiosis and microspore development (Downes and Marshall, 1971; Mamun et al., 2006; Wood et al., 2006; Gothandam et al., 2007; Sakata et al., 2014). Abnormal tapetum development and degradation under chilling stress results in aberrant pollen (Sakata et al., 2014). Plants insensitive to GA or deficient in GA production exhibit more severe problems in tapetal cell hypertrophy and pollen production under chilling. In the tapetum, chilling stress represses both the cell wall bound acid invertase gene *OSINV4* and the monosaccharide transporter gene *OSMST8*, which causes failed transport of sugar to the tapetum and developing pollen (Oliver et al., 2005; Mamun et al., 2006). ABA application also leads to abnormal pollen, possibly by repressing *OSINV4* and *OSMST8* (Oliver et al., 2005). More work needs to be done in the future to determine genes responsible for the abnormal development of tapetum under chilling conditions.

Later in development, anthesis and pollen germination are also cold sensitive. In wheat, chilling conditions [8/2°C (day/night)] applied to anthesis result in high levels of male sterility (Subedi et al., 1998). In *Arabidopsis*, freezing stress causes reduced pollen tube growth and decreased seed production. Similarly, mutations in G protein-coupled receptor-type G proteins (GTGs) lead to decreased pollen germination, abnormal pollen tube elongation, and consequent seed loss (Jaffé et al., 2012). In rice, the QTL *COLD1*, which encodes a regulator of G-protein signaling, acts to sense chilling (Ma et al., 2015). *COLD1* is important for maintaining grain yield, further suggesting that G-protein signaling plays a key role in chilling tolerance during sexual reproduction. In young rice panicles, the *Ctb1* QTL harbors an F-box protein gene that is responsible for chilling tolerance (Saito et al., 2010). Additionally, upregulation of the *CORN CYSTATIN* genes *CC8* and *CC9* is observed under cold stress (14 and 14/7°C). *CC8* is found in kernel and the immature tassel, while *CC9* is detected in immature and mature tassels, silk, and kernels (Massonneau et al., 2005). Future study looking into the roles of these cystatins in fertility could be valuable.

Collectively, similar to what is observed under heat stress, the stamen development is sensitive to cold stress, particularly during meiosis, tapetum development, pollen germination, and anthesis. The female organ development remains relatively unaffected to cold stress. However, not all studies agree with this finding. In maize, prolonged exposure to cold stress (10°C for 7 days) results in the abortion of the ear (Lejeune and Bernier, 1996). The effects of ear abortion may be prevented by applying benzyladenine (a synthetic cytokinin) exogenously (Lejeune et al., 1998). Genes like *COR* and those involved in GA and G-protein signaling may play important roles in chilling tolerance during sexual reproduction.

### Drought Stress

Similar to heat, drought stress affects flower development and consequently impairs fertility. In stamen development, drought stress causes shortened anther filaments, delayed anther development and dehiscence, as well as reduced pollen viability (Su et al., 2013; Tunc-Ozdemir et al., 2013; Ma et al., 2014). Female fertility is less sensitive to drought stress (Su et al., 2013). Younger buds are sacrificed during early drought stress and water is likely allocated to older flowers (Su et al., 2013).

Under moderate and severe drought stresses, thousands of genes are differentially expressed (Ma et al., 2014). Genes like *DREB1, ABA-RESPONSIVE ELEMENTS BINDING FACTORS* (*ABF), NAC DOMAIN CONTAINING PROTEIN019* (*NAC019*), *RESPONSIVE TO DESSICATION20* (*RED20*), and *RD29A* were upregulated (Su et al., 2013). A great number of genes involved in ABA and JA signaling are also upregulated, which may affect stamen filament elongation as well as overall stamen and pistil development (Su et al., 2013). *CYCLIC NUCLEOTIDE-GATED CHANNEL16* (*CNGC16*) is important for stress response, as disruption of *CHGC16* leads to reduced pollen viability (Tunc-Ozdemir et al., 2013).

Some cereals like rice and wheat are sensitive to drought stress, whereas others such as sorghum are quite drought tolerant. In maize, female organ development, particularly prior to pollination, is sensitive to drought stress, which is often attributed to problems with carbohydrate transport and metabolism. When comparing well watered with drought treated plants, carbohydrate transport to ovary is decreased in drought conditions and expression of carbohydrate (e.g., starch and sucrose) metabolism genes is altered (Mäkelä et al., 2005; Kakumanu et al., 2012). Many genes in maize kernels show differential expression under drought stress, such as those important for carbohydrate metabolism (*SU1P, ISA1, DULL1, FRK2, GLU1*, and *AAG1*), stress response and regulation (*ZmDJ1, SOD1*, and *STI1*), and transcriptional regulation of drought inducible genes (*EREBP1, MYB-IF35, MYB-IF25I*, and *RISBZ4*; Marino et al., 2009). Under drought conditions, genes involved in cell cycle, cell division, and antioxidant formation are down regulated in ovaries, while genes essential for stress responses like ABA are expressed at higher levels. Increased ABA may lead to a reduction of invertase in ovaries, limiting sucrose use and subsequent ovary abortion (Zinselmeier et al., 1999; Andersen et al., 2002; Boyer and Westgate, 2004; McLaughlin and Boyer, 2004; Kakumanu et al., 2012).

During meiosis, drought stress results in differential expression of many genes, for example, genes encoding β-carotene hydroxylase and cytochrome P450 monooxygenase which might protect against oxidative damage. Altered expression was also observed in genes that encode histone H2A and dehydrin DHN1, suggesting the importance of chromatin stabilization and dehydration prevention under drought stress (Zhuang et al., 2008). After pollination, elevated expression of senescence genes may be the cause of embryo abortion (McLaughlin and Boyer, 2004). Interestingly, pollen development was relatively unaffected by drought stress in maize (Schoper et al., 1986; Westgate and Boyer, 1986).

Drought stress is detrimental to pollen production in wheat, resulting in a 40–50% of reduction in yield (Dorion et al., 1996). Drought-induced degeneration of tapetal cells may contribute to the failure of microspore and pollen development. The timing of tapetum degeneration is crucial, as its early degeneration results in loss of orientation, and late degeneration leads to microspores that do not receive essential nutrients (Saini et al., 1984; Lalonde et al., 1997; Ji et al., 2010). In addition, pollen developed under drought condition is devoid of starch, limiting fertilization, and pollen tube growth (Ji et al., 2010). In wheat, drought stress decreases the level of invertases in developing pollen and microspores, (Dorion et al., 1996; Lalonde et al., 1997; McLaughlin and Boyer, 2004; Koonjul et al., 2005). Drought tolerant lines have a normal invertase expression (Ji et al., 2010). In wheat, no effects are observed on female fertility under moderate drought stress (Saini and Aspinall, 1981; Ji et al., 2010).

In rice, male sterility is common under drought stress conditions. If drought conditions are applied during PMC meiosis, the pollen production is severely affected (Sheoran and Saini, 1996), which is potentially caused by tapetal cell vacuolization/degeneration and abnormal starch deposition (Nguyen and Sutton, 2009; Jin et al., 2013). Under drought conditions, the presence of reactive oxygen species (ROS) results in a depletion of ATP and therefore leads to PCD and pollen abortion in rice (Nguyen et al., 2009). Furthermore, expression of genes critical for tapetal cell PCD and pollen wall formation is altered along with increased ABA signaling and decreased GA signaling (Jin et al., 2013). Both invertase and starch synthase gene expression are reduced under drought stress (Sheoran and Saini, 1996; Nguyen et al., 2010). Conversely, genes involved in sugar transport are upregulated (Sheoran and Saini, 1996; Nguyen et al., 2010; Fu et al., 2011). The accumulation of sugar may help maintain water levels in the anther due to low water potential (Fu et al., 2011).

**FIGURE 4:**
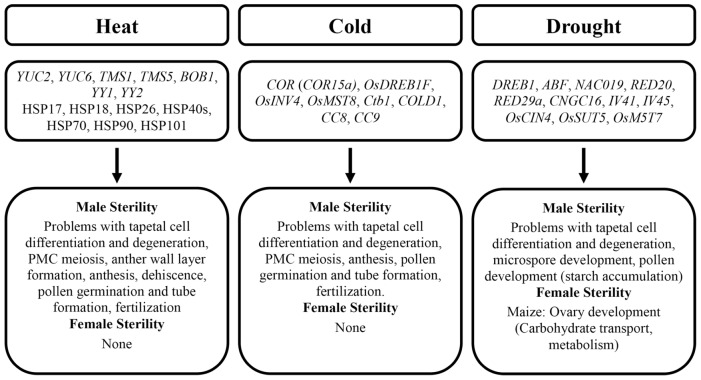
**Effects of abiotic stresses on fertility in *Arabidopsis* and key cereals.** Genes (italic) and proteins (non-italic) involved are shown.

It is not clear why the stamen development and male fertility appear more susceptible to abiotic stresses in plants. It will be necessary to identify genes that first respond to abiotic stresses and genes that later build strength for plants to cope with long-term abiotic stresses. Hormones, such as ABA and auxin, are heavily involved in abiotic stresses. It is well known that cross-talks are important for plant hormonal signaling; however, little is known about cross-talks among hormones in response to different abiotic stresses. The effects on fertility and some potential genes involved under abiotic stresses are summarized in **Table 2** and **Figure 4**.

**Table 2:** Effects of abiotic stresses on sterility and genes involved.

Stress	Organism	Key effects of stress	Genes involved
Heat	*Arabidopsis*	Abnormal microsporogenesis, irregular pollen and male sterility	*TMS1, BOB1, YUC2, YUC6*
Heat	Maize	Decreased pollen germination and pollen tube growth, decreased number of silks and florets, kernel abortion	*HSP70, HSP18, HSP101*
Heat	Wheat	Early tapetum degradation, decreased pollen viability, reduced anther size, irregular embryo sac development	*ARF, TPR*
Heat	Rice	Abnormal microsporogenesis, decreased pollen production and viability, altered flower timing	*YY1, YY2*
Chilling	*Arabidopsis*	Decreased pollen viability and pollen tube growth	*COR, WRKY*, C*BF1*
Chilling	Maize	Decreased number of tassel branches and spikelet pairs, decreased pollen production, ear abortion	*CC8, CC9*
Chilling	Wheat	Pollen death and male sterility, no affects on female development	None
Chilling	Rice	Abnormal microspore, pollen development, and tapetum degradation	*COLD1, OsINV4, OsMST8, OsDREB1F*
Drought	*Arabidopsis*	Decreased anther filament length, delays in anther development and dehiscence, decreased pollen viability	*DREB1, ABF, NAC019, RED20, RD29A, CNGC16*
Drought	Maize	Decreased number of kernels and increased embryo abortion	*TM00030371, TM00036151, H2A, DHN1, SIP1, SU1P, ISA1, DULL1, FRK2, GLU1, AAG1, ZmDJ1, SOD1, STI1, EREBP1, MYB-IF35, MYB-IF25I, RISBZ4*
Drought	Wheat	Abnormal microspore and tapetum development, pollen devoid of starch, decreased fertilization and pollen tube growth	*IVR1, IVR5*
Drought	Rice	Decreased pollen viability, abnormal tapetal degeneration and starch deposits in pollen	*OsDREB1F, Os*miR408, *OsCIN4, OsSUT5, OsM577*

## Conclusion and Future Directions

Floral organ degeneration or abortion under the normal condition results in the formation of unisexual flowers, such as in maize; or completely sterile flowers, such as in sorghum. In addition to other genes, class B and C genes are involved in floral reproductive organ degeneration via losing their functions in floral organ identity or in regulating expression of downstream target genes. Moreover, hormones play important roles in establishing the male and female state. Genes underlying JA and BR signaling and their biosynthesis promote stamen development and carpel abortion, whereas genes involved in GA signaling and their biosynthesis induce carpel development and stamen abortion (with exception of the maize ear). Auxin is essential for the formation of all floral organs, including stamen and carpels. Interactions between flower and hormone regulation genes are essential for flower organ establishment and fertility.

It is evident that maintaining ideal temperature and soil moisture is crucial for fertility in *Arabidopsis*, maize, wheat, and rice. Abiotic stresses commonly lead to male sterility, while female viability is well maintained under most mild abiotic stresses. In nearly all plants under all observed abiotic stresses, the most sensitive stages causing sterility are during tapetum development, male meiosis, microsporogenesis, anthesis, and fertilization. Hormones play important roles in male and female organ development during abiotic stresses. Auxin application can reverse some effects of heat stress, whereas decreased GA in stressed plants worsens tapetum defects and consequently further reduces pollen production.

Overall, abiotic stress induced sterility causes the major loss of crop yield. By 2050 the global population is expected to reach 9.1 billion. Additionally, if the use of grains for biofuel production is intensified, the demand for crop products will be further increased. High-yield wheat ideotypes are currently being studied and improved based on long-term climate projections (Semenov and Stratonovitch, 2013). To develop high-yield crops that have ideal agronomic traits and can cope with anticipated environmental changes using traditional and molecular breeding approaches, it is necessary to decipher molecular genetic mechanisms that cause flower sterility under normal and stress conditions.

## Author Contributions

AS and DZ conceived the idea and wrote the manuscript.

## Conflict of Interest Statement

The authors declare that the research was conducted in the absence of any commercial or financial relationships that could be construed as a potential conflict of interest.
